# Supplementary material for: Development of LAT1-Selective Nuclear Medicine Therapeutics Using Astatine-211
Source: Int J Mol Sci. 2024 Nov 18;25(22):12386. doi: 10.3390/ijms252212386 (PMC11594329; doi:10.3390/ijms252212386)
Supplement: Supplementary file 1 [file ijms-25-12386-s001.zip › ijms-3308704-supplementary.pdf]

International Journal of *Molecular Sciences*

MDPI

Development of LAT1-selective nuclear medicine therapeutics using astatine-211

Kazuko Kaneda-Nakashima [1, 2*], Yoshifumi Shirakami [2], Kentaro Hisada [1], Sifan Feng [1], Yuichiro Kadonaga [2], Kazuhiro Ooe [2], Tadashi Watabe [3], Yoshiyuki Manabe [2, 4], Atsushi Shimoyama [2, 4], Masashi Murakami [2], Atsushi Toyoshima [2], Hiromitsu Haba [5], Yoshikatsu Kanai [6], Koichi Fukase [2, 4]

Supplemental method

S1. Hematological analysis

S1.1. Animal preparation and administration of ^{211}At-AAMT-O-Me-L

This study was conducted in compliance with the Animal Experiment Regulations and the Act on Welfare and Management of Animals of the Osaka University Graduate School of Science (approval numbers: 2020-02-0). Normal male ICR mice (n = 24, 5 weeks old, body weight: 26.09 ± 0.12 g) and normal female ICR mice (n = 24, 5 weeks old, body weight: 23.14 ± 0.01 g) were purchased from Japan SLC Inc. (Hamamatsu, Japan), respectively. The animals were housed under a 12 h light/12 h dark cycle and acclimated for one week prior to use. General condition and body weight were monitored. ^{211}At-AAMT-O-Me-L was administered intravenously to 24 mice. For the toxicity study, three doses of ^{211}At-AAMT-O-Me-L (1MBq/mouse, approximately 50 MBq/kg, single dose) or saline were intravenously administered for the main evaluation point on day 1, 5, 14 and 28 days.

S1.2. Blood Examination of normal animals

After the prescribed observation time, blood was collected under anesthesia and the animals were euthanized. Organs were harvested, fixed and preserved for pathological analysis. A blood cell counter was used for the measurements (CB-1010; ARKRAY, Inc., Kyoto, Japan). Red blood cell count (RBC), hemoglobin concentration (Hb), hematocrit (Ht), white blood cell count (WBC), platelet count (platelet), lymphocytes (%Lymph), monocytes (%Mon), and granulocytes (%Gra) were calculated (**Supplementary Table S2**).

Supplemental data

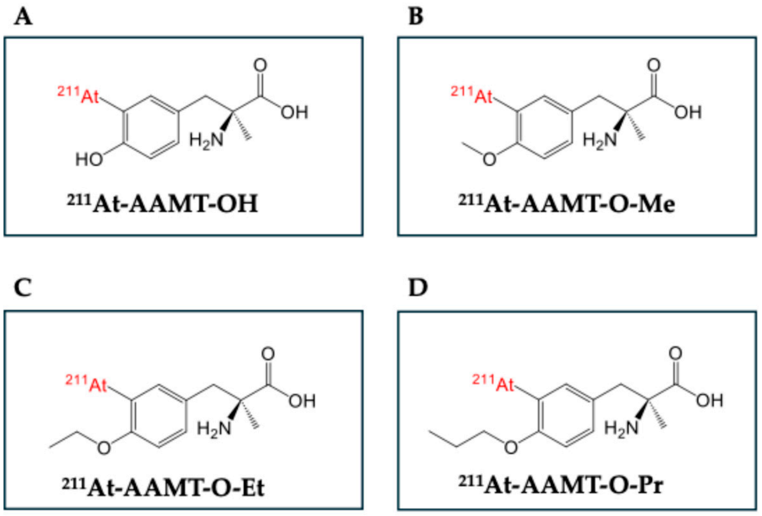

Supplementary Figure S1. Structure of experimental labeling compounds.

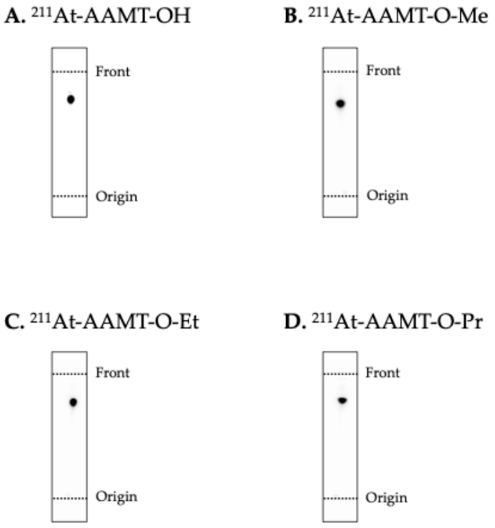

Supplementary Figure S2. Evaluation of experimental labeling compounds by TLC. All compounds were labeled using *Shirakami Reaction*, purified using an HLB column before TLC analysis.

Supplementary Table S1. Urinary excretion rate (% ID)

Strain	Compound	1h	3h
ICR	^{211}At-AAMT-O-Me-D	24.84 ± 13.03	71.36 ± 4.67
	^{211}At-AAMT-O-Me-L	33.69 ± 1.26	23.99 ± 2.40
PANC1/Nude	^{211}At-AAMT-O-Me-D	35.24 ± 8.70	47.38 ± 3.19
	^{211}At-AAMT-O-Me-L	20.40 ± 0.76	3.23 ± 0.67

Supplementary Table S2. Hematology - Single dose intravenous toxicity study of ^{211}At -AAMT - OMe-L in mice -

RBC: Red blood cell; Hb: Hemoglobin; Ht: Hematocrit; MCV: Mean corpuscular volume; MCH: Mean corpuscular hemoglobin; MCHC: Mean corpuscular hemoglobin concentration; WBC: White blood cell; Lym: Lymphocyte; Mon: Monocyte; Gra: Granule. Significantly different from the control, * p<0.05, ** p<0.01, *** p<0.001. (t-test)

Inspection time	Sex	Group		RBC ($\times10^6$ cells/μL)	Hb (g/dL)	Ht (%)	MCV (fL)	MCH (pg)	MCHC (g/dL)	WBC ($\times10^3$ cells/μL)	Platelet ($\times10^3$ cells/μL)	%Lymph (%)	%Mon (%)	%Gra (%)
1 days after administration	Male	Control	N=3	9.23 ± 0.05	16.73 ± 0.29	49.00 ± 0.29	53.13 ± 1.14	18.06 ± 0.33	34.13 ± 0.01	17.30 ± 3.11	230.00 ± 27.59	60.63 ± 2.78	7.70 ± 0.81	31.67 ± 2.18
		50 MBq/kg	Mean ± S.E.	8.43 ± 0.17*	14.73 ± 0.29	44.67 ± 0.29*	53.03 ± 0.31	17.43 ± 0.67	32.93 ± 0.04*	14.00 ± 2.45	151.33 ± 72.46	64.30 ± 2.70	4.93 ± 0.48*	30.77 ± 2.22
	Female	Control	N=3	8.59 ± 0.12	15.27 ± 0.45	45.43 ± 1.32	52.97 ± 1.07	17.73 ± 0.32	33.23 ± 0.52	11.77± 1.73	161.67± 12.71	62.45 ± 0.35	7.30 ± 0.57	30.23± 0.82
		50 MBq/kg	Mean ± S.E.	7.70 ± 0.53	13.20 ± 0.95	39.53 ± 2.84	51.40 ± 0.44	17.10 ± 0.17	56.86 ± 0.03	12.37 ± 1.24	214.00 ± 47.44	64.50 ± 0.57*	5.73 ± 0.19	29.77 ± 0.75
5 days after administration	Male	Control	N=3	7.64 ± 0.16	128.00 ± 2.00	40.07 ± 0.75	52.50 ± 0.60	16.70 ± 0.10	31.93 ± 3.18	16.80 ± 0.75	809.00 ± 136.15	10.50 ± 2.13	1.03 ± 0.19	5.27 ± 2.67
		50 MBq/kg	Mean ± S.E.	6.85 ± 0.23*	119.00 ± 5.57	36.43 ± 1.69	53.23 ± 0.78	17.33 ± 0.24	32.60 ± 1.73	4.33 ± 1.00***	379.00 ± 76.00*	3.10 ± 4.05***	0.20 ± 0.06*	1.03 ± 3.84*
	Female	Control	N=3	7.31 ± 0.22	12.96 ± 3.28	39.43 ± 1.39	54.00 ± 0.87	17.67 ± 0.09	32.86 ± 4.81	12.97 ± 1.91	424.00 ± 31.09	7.97 ± 4.25	0.83 ± 0.15	4.17 ± 4.39
		50 MBq/kg	Mean ± S.E.	6.85 ± 0.39	11.57 ± 4.91	35.67 ± 2.23	52.13 ± 0.28	16.90 ± 0.26	32.47 ± 6.57	2.67 ± 0.97**	508.33 ± 39.43*	1.73 ± 1.64*	0.17 ± 0.07*	0.77 ± 1.21*
14 days after administration	Male	Control	N=3	10.20 ± 0.51	173.33 ± 7.97	50.53 ± 2.44	49.27 ± 0.75	16.97 ± 0.27	345.00 ± 2.00	6.33 ± 1.23	675.33 ± 16.15	72.13 ± 2.76	1.97 ± 0.19	25.90 ± 2.59
		50 MBq/kg	Mean ± S.E.	9.29 ± 0.64	165.33 ± 11.46	48.33 ± 3.19	52.13 ± 0.15	17.73 ± 0.09	341.33 ± 2.19	5.77 ± 2.19	176.33 ± 2.94	52.30 ± 6.08*	8.70 ± 1.36**	39.00 ± 4.87
	Female	Control	N=3	10.32 ± 0.43	181.00 ± 8.39	51.83 ± 2.45	50.30 ± 1.36	17.50 ± 0.40	348.67 ± 1.20	4.90 ± 0.46	627.33 ± 27.54	78.97 ± 1.65	2.70 ± 0.35	18.33 ± 1.33
		50 MBq/kg	Mean ± S.E.	9.34 ± 0.42	163.00 ± 4.04	47.50 ± 1.69	54.27 ± 3.21	17.43 ± 0.35	343.00 ± 4.04	5.63 ± 2.64	264.00 ± 2.72*	59.40 ± 5.86*	7.53 ± 1.65*	33.07 ± 4.29*
28 days after administration	Male	Control	N=3	9.74 ± 0.28	16.37 ± 0.42	49.40 ± 1.89	50.77 ± 0.66	16.77 ± 0.12	33.10 ± 0.40	7.50 ± 0.79	552.00 ± 54.52	67.43 ± 9.78	5.23 ± 3.28	27.33 ± 6.71
		50 MBq/kg	Mean ± S.E.	9.13 ± 0.25	15.20 ± 0.42	45.43± 1.58	49.83 ± 0.96	16.57 ± 0.08	33.43 ± 0.57	7.47 ± 1.28	359.00 ± 42.35*	68.30 ± 11.06	5.20 ± 3.00	26.50 ± 8.07
	Female	Control	N=3	8.59 ± 0.72	14.37 ± 0.49	43.50 ± 4.29	50.50 ± 0.78	16.63 ± 0.32	32.93 ± 0.18	4.13 ± 0.62	537.00 ± 96.91	69.27 ± 7.32	6.07 ± 4.21	24.67 ± 3.14
		50 MBq/kg	Mean ± S.E.	8.19 ± 0.27	13.83 ± 1.46	41.53 ± 0.89	50.80 ± 0.62	16.87± 0.42	33.26 ± 0.49	6.97 ± 1.88	609.00 ± 117.18	70.03 ± 3.01	3.47 ± 0.23	26.50 ± 2.86
